# Adolescent cocaine exposure simplifies orbitofrontal cortical dendritic arbors

**DOI:** 10.3389/fphar.2014.00228

**Published:** 2014-10-27

**Authors:** Lauren M. DePoy, Riley E. Perszyk, Kelsey S. Zimmermann, Anthony J. Koleske, Shannon L. Gourley

**Affiliations:** ^1^Department of Pediatrics, Emory University School of Medicine, Atlanta, GA, USA; ^2^Yerkes National Primate Research Center, Emory University, Atlanta, GA, USA; ^3^Graduate Program in Neuroscience, Emory University, Atlanta, GA, USA; ^4^Graduate Program in Molecular and Systems Pharmacology, Emory University, Atlanta, GA, USA; ^5^Department of Molecular Biophysics and Biochemistry, Yale University, New Haven, CT, USA; ^6^Interdepartmental Neuroscience Program, Yale University, New Haven, CT, USA; ^7^Department of Neurobiology, Yale University School of Medicine, New Haven, CT, USA

**Keywords:** psychostimulant, orbital, sholl, adolescence, addiction

## Abstract

Cocaine and amphetamine remodel dendritic spines within discrete cortico-limbic brain structures including the orbitofrontal cortex (oPFC). Whether dendrite structure is similarly affected, and whether pre-existing cellular characteristics influence behavioral vulnerabilities to drugs of abuse, remain unclear. Animal models provide an ideal venue to address these issues because neurobehavioral phenotypes can be defined both before, and following, drug exposure. We exposed mice to cocaine from postnatal days 31–35, corresponding to early adolescence, using a dosing protocol that causes impairments in an instrumental reversal task in adulthood. We then imaged and reconstructed excitatory neurons in deep-layer oPFC. Prior cocaine exposure shortened and simplified arbors, particularly in the basal region. Next, we imaged and reconstructed orbital neurons in a developmental-genetic model of cocaine vulnerability—the *p190rhogap*+/– mouse. p190RhoGAP is an actin cytoskeleton regulatory protein that stabilizes dendrites and dendritic spines, and *p190rhogap*+/– mice develop rapid and robust locomotor activation in response to cocaine. Despite this, oPFC dendritic arbors were intact in drug-naïve *p190rhogap*+/– mice. Together, these findings provide evidence that adolescent cocaine exposure has long-term effects on dendrite structure in the oPFC, and they suggest that cocaine-induced modifications in dendrite structure may contribute to the behavioral effects of cocaine more so than pre-existing structural abnormalities in this cell population.

## INTRODUCTION

Cocaine addiction is characterized by maladaptive decision making, a loss of control over drug consumption, and habit-like drug seeking despite adverse consequences. These cognitive changes likely reflect the effects of repeated drug exposure on prefrontal cortical neurobiology that then further promote drug use (Jentsch and Taylor, 1999; Everitt and Robbins, 2005; Torregrossa et al., 2011; Lucantonio et al., 2012). Additionally, *pre-existing* neurobehavioral characteristics in drug-naïve individuals may contribute to drug vulnerabilities (Ersche et al., 2012). Rodents provide an ideal model system to isolate vulnerability factors in drug-naïve organisms and also characterize the *consequences* of cocaine exposure because like humans, rodents will readily self-administer cocaine and engage in complex decision making, as well as relapse-like behavior. Also as in humans, individual differences in behavioral response strategies can serve as phenotypic predictors of addiction-like behaviors such as drug seeking following periods of abstinence (Deroche-Gamonet et al., 2004). Finally, even experimenter-administered, rather than self-administered, cocaine can induce behavioral phenotypes in rodents that are relevant to addiction etiology in humans, e.g., increased propensity to engage in reward-seeking habits (Schoenbaum and Setlow, 2005; Gourley et al., 2013c; Hinton et al., 2014).

We recently sought to identify the long-term consequences of cocaine exposure during adolescence, when drug use is often initiated in humans, and when the prefrontal cortex is still developing (Casey et al., 2000; Giedd, 2004; Paus et al., 2008). We found that >7 weeks following subchronic exposure during adolescence, cocaine-exposed mice preferentially engaged habit-like response strategies at the expense of flexible action-outcome-based strategies to acquire food reinforcers (Hinton et al., 2014). Inactivation of the orbitofrontal cortex (oPFC) blocks goal-directed action selection in the same task (Gourley et al., 2013a), and adolescent cocaine exposure eliminates dendritic spines in this region (Gourley et al., 2012). Whether adolescent cocaine exposure regulates oPFC dendrite arbor structure is, to our knowledge, unresolved. Such gross remodeling could contribute to persistent maladaptive decision making following adolescent cocaine exposure and potentially to increased risk of dependence (O’Brien and Anthony, 2005) or decreased likelihood of seeking treatment (Kessler et al., 2001) in individuals who initiate cocaine use in adolescence.

We first quantified the effects of adolescent cocaine exposure on behavioral flexibility in an oPFC-dependent instrumental reversal task and on dendrite arbor structure in adult excitatory deep-layer oPFC neurons. Next, we aimed to evaluate whether *pre-existing* morphological abnormalities in the same neuron population were associated with behavioral vulnerabilities. The model we selected for this experiment was the *p190rhogap*+/– mouse. These mice are deficient in p190RhoGAP, a principal Src substrate in the brain that regulates cell structure through interactions with the RhoA GTPase (Brouns et al., 2000, 2001). Drug-naïve *p190rhogap*+/– mice appear at baseline to be behaviorally unremarkable (Gourley et al., 2012, 2013b). Nonetheless, they are highly sensitive to cocaine such that a single injection elicits a sensitization-like response (Gourley et al., 2012), making them an ideal candidate model by which to isolate pre-existing structural factors associated with subsequent cocaine vulnerability.

Our findings suggest that adolescent cocaine exposure simplifies excitatory oPFC dendritic arbors. By contrast, dendrite arbors appear grossly normal in *p190rhogap*+/– mice and thus, do not obviously account for behavioral vulnerabilities to cocaine in these animals.

## MATERIALS AND METHODS

### SUBJECTS

All mice were bred on a C57BL/6 background, and those used in anatomical studies expressed *thy1*-derived yellow fluorescent protein (YFP; Feng et al., 2000) to enable dendrite imaging. YFP-expressing p190RhoGAP-deficient mice (*p190rhogap*+/–) or *p190rhogap*+/+ littermates were also used. *p190rhogap*+/– mice have a ~32–40% reduction in p190RhoGAP protein expression (Brouns et al., 2000). Mice were maintained on a 12-h light cycle (0700 on), and provided food and water *ad libitum* unless otherwise noted. Procedures were in accordance with the *Guide for the Care and Use of Laboratory Animals* and approved by the Emory and Yale University Institutional Animal Care and Use Committees, as appropriate.

### ADOLESCENT COCAINE EXPOSURE

Cocaine or saline (Sigma) was administered for five consecutive days starting at postnatal day (P) 31 (10 mg/kg, i.p., 1 ml/100 g). Then, mice were left undisturbed until P56, at which point they were euthanized for anatomical studies or tested in an instrumental reversal learning task.

### INSTRUMENTAL REVERSAL LEARNING

Mice with a history of adolescent cocaine exposure were food-restricted as adults to ~93% of their original body weight and trained to nose poke for food reinforcement (20 mg grain-based pellets; Bioserv) using illuminated Med-Associates conditioning chambers. Training was initiated with a continuous reinforcement schedule; 30 pellets were available for responding on each of two distinct nose poke recesses located on opposite sides of a single wall within the chambers, resulting in 60 pellets/session. Sessions ended when all 60 pellets were delivered or at 135 min. Responding on a center aperture was not reinforced. Following 7 days of training, mice were required to “reverse” their responding to this center aperture to continue to obtain reinforcement. Responding on the previously active apertures was no longer reinforced. These “reversal” sessions were 25 min in duration and used a variable ratio two schedule of reinforcement (Gourley et al., 2010). Responses on the active and inactive nose poke apertures were quantified, as were head entries into the magazine where pellets were delivered. One cocaine-exposed mouse consistently generated values two standard deviations above the group mean and was excluded.

### LOCOMOTOR MONITORING

In 8-week-old mice, we used a within-subjects design to compare the locomotor response to cocaine between *p190rhogap*+/– and *p190rhogap*+/+ littermates. Mice were administered cocaine (10 mg/kg, i.p., 1 ml/100 g) for five sequential days, and then left undisturbed for 7–10 days at which point a “challenge” injection was administered (10 mg/kg, i.p., 1 ml/100 g).

Locomotor activity was monitored using customized Med-Associates chambers equipped with 16 photobeams. Mice were first habituated to the chambers for 1 h following a saline injection, then cocaine was administered. Total photobeam breaks following the cocaine injection were normalized to those generated in the 30 min following saline injection. During the challenge session, all mice were habituated to the locomotor monitoring chambers for 1 h without injection, then saline was administered and mice were monitored for 30 min, and finally, cocaine was administered, and mice were monitored for an additional 30 min. Cocaine-elicited photobeam breaks were normalized to those following saline in order to control for conditioned locomotor activation in response to injection. This experiment served to provide evidence that *p190rhogap*+/– mice are hyper-sensitive to cocaine, as we have previously reported (Gourley et al., 2012), but importantly, mice used for anatomical studies were cocaine-naïve because our goal was to evaluate pre-existing factors that might be associated with cocaine vulnerability.

### DENDRITIC ARBOR RECONSTRUCTION AND MEASUREMENT

Mice were euthanized, and fresh brains were submerged in 4% paraformaldehyde for 48 h, then transferred to 30% w/v sucrose, followed by sectioning into 50 µm-thick coronal sections on a microtome held at –15°C. These relatively thin sections allow us to image whole deep-layer neurons without background fluorescence that would otherwise obstruct reconstruction. Little is known regarding the typical morphology of these neurons. This may be because traditional Golgi impregnation can spare deep-layer oPFC (Kolb et al., 2008), and while it is conceivable that we under-count dendrites that may have been truncated along the rostro-caudal plane, neurons with clear dendritic arbor truncations were excluded from the analyses.

Neurons were imaged on a spinning disk confocal (VisiTech International, Sunderland, UK) on a Leica microscope. Z-stacks were collected with a 20 × 1.4 NA objective using a 1-µm step size, sampling above and below the neuron. After imaging, we confirmed at 10 × that the image was collected from the oPFC. Most images were collected from the lateral oPFC, however, the ventral subregion was also sampled. Neurons contained at least two basal dendritic arbors and a distinct intact apical dendrite, all with at least second-order branching.

Neurons were reconstructed in three dimensions by a single experimenter blind to group using Neurolucida (MBF Biosciences). Total dendritic material was measured for apical and basal arbors. To assess dendrite complexity, a 3-D version of a Sholl analysis (Wellman et al., 2007) was performed by measuring the number of dendritic intersections within 10-µm concentric spheres radiating from the soma. Four to 11 neurons/mouse from the *p190rhogap*+/– population and four to eight neurons/mouse from the cocaine-exposed population were imaged, reconstructed, and analyzed. Group sizes were six to seven mice in the cocaine-exposed population and four to six mice in the *p190rhogap*+/– population.

### STATISTICAL ANALYSES

For instrumental conditioning studies, responding on the active and inactive apertures and magazine head entry rates were compared by ANOVA with repeated measures and group as the independent variable. For locomotor assessments, cocaine-elicited photobeam breaks (calculated as potentiation from baseline) were compared by ANOVA with repeated measures and group as the independent variable. Locomotor counts on the challenge day were compared between groups by unpaired t-test.

For anatomical studies, each mouse contributed a single value—the mean of its multiple neurons—to the analyses. Dendrite lengths were compared between groups by unpaired t-test. Sholl intersections were compared by ANOVA with repeated measures. In the case of interactions, *post hoc* comparisons were generated using Tukey’s t-tests; the results of *post hoc* comparisons are indicated graphically. *p* < 0.05 was considered significant. In one instance, a Kolmogorov–Smirnov comparison was also applied to total dendrite length; in this case, each neuron was considered an independent sample.

## RESULTS

Here we aimed to quantify the effects of adolescent cocaine exposure on oPFC dendrite morphology. We first, however, confirmed that adolescent cocaine had long-term behavioral consequences. Mice were exposed to subchronic cocaine during early adolescence, from P31 to P35 (Spear, 2000), then left undisturbed until adulthood. Cocaine exposure is thought to confer a bias toward inflexible, maladaptive decision-making strategies, so we tested mice in an instrumental “reversal learning” task that is sensitive to chronic cocaine exposure or lesions of the oPFC in adult mice (Krueger et al., 2009; Gourley et al., 2010).

**FIGURE 1:**
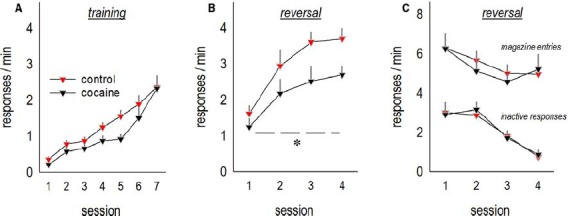
**Adolescent cocaine exposure impairs instrumental reversal learning in adulthood. (A)** Mice were exposed to cocaine or saline from P31 to P35, then left undisturbed until adulthood, at which point they were trained to nose poke for food reinforcers. **(B)** When the location of the reinforced aperture within the chamber was then “reversed,” cocaine-exposed mice generated fewer responses on the now-active aperture. **(C)** By contrast, cocaine exposure did not impact response inhibition on the previously reinforced apertures or head entries into the food-associated magazine. Means + SEMs, *p < 0.05, main effect of cocaine.

Mice with a history of subchronic saline or cocaine exposure were able to acquire a nose poke response for food reinforcement as adults (interaction F < 1; Figure 1A). Qualitatively, cocaine appeared to modestly decrease overall responding, but this effect did not reach significance [main effect *F*_(1,21)_ = 3.9, *p* = 0.06], and response rates were equivalent at the end of training. When the response requirement was “reversed” such that mice were required to respond on an aperture at a separate location in the conditioning chamber, mice with a history of cocaine exposure generated fewer responses [main effect of cocaine *F*_(1,20)_ = 4.1, *p* < 0.05; Figure 1B]. We identified no effects of cocaine on responding on the previously reinforced aperture or magazine head entry rate (both F < 1; Figure 1C). This pattern recapitulates the effects of prolonged cocaine exposure in adult mice (Krueger et al., 2009), as well as lesions of the lateral oPFC (Gourley et al., 2010).

**FIGURE 2:**
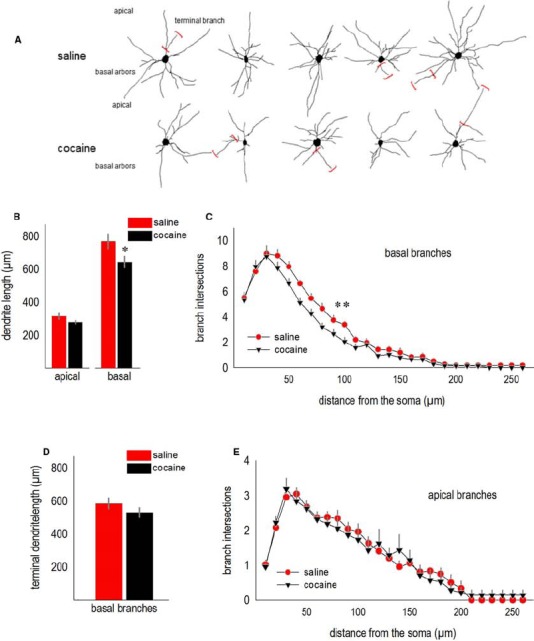
**Cocaine exposure during adolescence simplifies oPFC dendrite arbors in adulthood. (A)** Representative deep-layer oPFC pyramidal neurons are shown. Terminal branches are indicated by red brackets. **(B)** Total dendrite length in the apical tree was not significantly affected, but basal lengths were reduced. **(C)** Simultaneously, dendritic arbors on the basal tree were simplified, as indicated by fewer Sholl intersections 40–100 µm from the soma. **(D)** The total length of basal terminal branches did not significantly differ between groups, consistent with arbor simplification in relatively close proximity to the soma in **(C)**. **(E)** Sholl intersections for apical arbors did not differ. Means + SEMs. *p < 0.05 vs. saline, **p < 0.05 for 40–100 µm from the soma.

We next analyzed the effects of adolescent cocaine exposure on dendrite structure in the adult lateral oPFC. Representative deep-layer neurons from adult mice are shown (Figure 2A). Note the relatively stellate shape of oPFC pyramidal neurons compared to the more classically pyramidal shape of neurons in other subregions of the prefrontal cortex (further discussed in Kolb et al., 2008; see also Liston et al., 2006; Bortolato et al., 2011).

Total basal arbor length was reduced in cocaine-exposed mice (*t*11 = 2.3, *p* < 0.05; Figure 2B). Basal arbors were also less complex, as indicated by fewer Sholl intersections 40–100 µm from the soma [interaction *F*_(25,275)_ = 1.9, *p* < 0.01; Figure 2C]. As another metric of dendrite length, we measured terminal branches, the segments following the last bifurcations of each dendrite. In this case, terminal branch lengths did not differ (*t*11 = –1.3, *p* > 0.2; Figure 2D), consistent with evidence from the Sholl analysis that a history of adolescent cocaine exposure simplifies dendrite arbors in close proximity to the soma (again, Figure 2C).

Despite differences in basal arbor length and complexity, apical arbors were not significantly affected (for length, *t*11 = 1.9, *p* = 0.09; for Sholl intersections, Fs < 1; Figures 2B,E).

**FIGURE 3:**
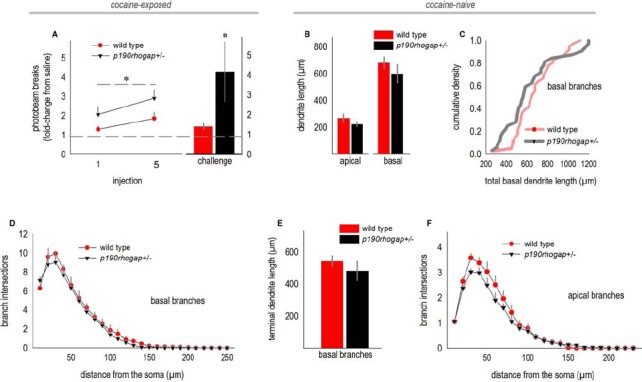
**oPFC dendrites in drug-naïve *p190rhogap*+/– mice are intact. (A)** *p190rhogap*+/– mice are hyper-sensitive to cocaine, as indicated by increased locomotor activity following cocaine injection, relative to saline injection (dashed line at 1), and relative to littermate wild type mice. The bar graph shows that following a washout period, cocaine injection elicited greater photobeam breaks in *p190rhogap*+/– mice than in wild type littermates. **(B)** We next assessed the morphology of deep-layer oPFC neurons in drug-naïve mice. Length of the apical and basal dendrites did not differ between drug-naïve groups, despite behavioral vulnerabilities to cocaine. **(C)** Basal dendrites are represented another way—here, total dendrite length for each neuron is represented in a cumulative density function. Again, we identified no significant differences between groups. **(D)** Sholl analyses of basal arbors also revealed no differences in complexity. **(E)** Additionally, the length of the terminal arbors did not differ. **(F)** Sholl analyses indicated that the complexity of apical arbors also did not differ. Means + SEMs. *p ≤ 0.05 vs. wild type.

We next evaluated dendrite complexity in a developmental-genetic model of cocaine vulnerability—*p190rhogap*+/– mutant mice. We selected these mice because p190RhoGAP is a cytoskeleton regulatory protein implicated in postnatal dendrite stability in the brain (Sfakianos et al., 2007), and we have previously reported that *p190rhogap*+/– mice display augmented sensitivity to cocaine (Gourley et al., 2012). Specifically, mice develop a sensitization-like response even after exposure to a single relatively low dose, and locomotor activity remains exaggerated over the course of several daily cocaine administrations, an effect that we recapitulate here by administering 10 mg/kg cocaine to mice daily for 5 days [main effect of genotype *F*_(1,20)_ = 7.4, *p* = 0.01; Figure 3A]. Mice were then left undisturbed for 1 week, after which they were habituated to the locomotor monitoring chambers for 1 h, then injected with saline and monitored for 30 min, then finally, injected with cocaine. Wild type mice generated 1.4 times as many photobeam breaks following low-dose cocaine “challenge” relative to saline. By contrast, *p190rhogap*+/– littermates broke >4-fold more photobeams following cocaine exposure (*t*19 = –2.1, *p* = 0.05; Figure 3A).

Adult drug-naive *p190rhogap*+/– mice were crossed with mice expressing YFP, generating YFP-expressing wild type and *p190rhogap*+/– offspring, and allowing us to potentially identify structural predictors of cocaine vulnerable *prior to drug exposure*. When oPFC dendrites from drug-naïve *p190rhogap*+/– mice were imaged and reconstructed, however, we identified no differences in total dendrite length (apical, *t*8 = 1.2, *p* = 0.3; basal, *t*8 = 0.8, *p* = 0.5; Figure 3B). By contrast, basal dendrite lengths differed in *cocaine-exposed* mice above, so as an additional, potentially more nuanced measure, we compared dendrite lengths using a Kolmogorov–Smirnov analysis in which the basal dendrite length from each neuron was considered an independent sample. Even here, we again did not identify differences between wild type and *p190rhogap*+/– mice (*D* = 0.2, *p* = 0.3; Figure 3C). Consistent with this outcome, basal arbor complexities did not differ, as determined by Sholl intersections (interaction F < 1; Figure 3D), and the length of the terminal branches did not differ (*t*8 = –1, *p* = 0.3; Figure 3E).

When we quantified Sholl intersections for the apical arbor, neither interactions nor main effects were detected [interaction F = 1; main effect *F*_(1,22)_ = 1.4, *p* = 0.3; Figure 3F]. Qualitatively, wild type mice appeared to have more complex arbors, but this impression was driven by a single mouse.

## DISCUSSION

The ability of neurons to integrate into networks and regulate behavior is determined in part by the size, shape, and complexity of dendrites. Dendrites can be remarkably plastic—for example, oPFC dendritic arbors remodel following stressor exposure (Liston et al., 2006; Dias-Ferreira et al., 2009) and environmental enrichment (Comeau et al., 2010). Some such modifications may play a role in mood disorders and other psychopathologies involving cortico-striatal circuits (e.g., cocaine addiction), but the characterization of structural modifications that—like drug craving in addiction—persist beyond the period of active drug exposure remains incomplete. We used transgenic mice expressing *thy1*-derived YFP to isolate and reconstruct dendritic arbors of excitatory deep-layer oPFC neurons. We report that arbors remodeled in response to subchronic cocaine exposure in adolescence and were simplified in adulthood. By contrast, dendritic arbors in drug-naïve *p190rhogap*+/– mutant mice—a model of cocaine vulnerability (Gourley et al., 2012)—were intact, suggesting that the *response* to cocaine, rather than pre-existing structural deficiencies *per se*, is associated with behavioral sensitivity to further drug exposure in these mice.

### PREFRONTAL CORTICAL DENDRITES REORGANIZE IN RESPONSE TO COCAINE

The effects of amphetamine-like psychostimulants such as cocaine on neural structure have been intensively studied since the seminal reports of Robinson and Kolb (1997, 1999) describing drug-induced dendrite and dendritic spine elaboration in the nucleus accumbens and medial prefrontal cortex. Within the prefrontal cortex, the vast majority of subsequent research has remained focused on medial wall structures, largely sparing the oPFC; this is despite overwhelming evidence implicating oPFC function in addiction etiology (e.g., see Lucantonio et al., 2012). Currently available data indicate that amphetamine and cocaine *reduce* dendritic spine density in the oPFC (Kolb et al., 2004; Crombag et al., 2005; Muhammad and Kolb, 2011a,b; Gourley et al., 2012; but see Ferrario et al., 2005), but effects on dendrite structure remain unclear. We report novel evidence that cocaine exposure simplifies oPFC dendrite arbors, particularly in the basal region. Notably, chronic ethanol exposure does not remodel excitatory oPFC neurons (Holmes et al., 2012; DePoy et al., 2013), thus the present effects may be selective to stimulants, or potentially cocaine specifically.

In the parietal cortex, amphetamine exposure blocks the dendrite-elaborating effects of environmental enrichment, consistent with our current findings, although amphetamine alone has no consequences (Kolb et al., 2003). Nonetheless, we found evidence of long-term dendrite *simplification* following cocaine. How might we reconcile this apparent contradiction? One difference, in addition to the anatomical, is that cocaine was administered here during the equivalent of adolescence, a period of vulnerability to the development of dependence in humans (Anthony and Petronis, 1995; O’Brien and Anthony, 2005). Recent studies using small-animal magnetic resonance imaging complement ours, revealing that adolescent (though not adult) cocaine exposure results in cortical thinning (Wheeler et al., 2013). Interestingly, overall oPFC volume is *increased* following adolescent cocaine exposure; this increase could conceivably reflect glial responses to cocaine (Bowers and Kalivas, 2003; Haydon et al., 2009), though further investigations are necessary.

Here, oPFC neurons in mice with a history of adolescent cocaine exposure were simplified, particularly in the basal region. This is notable given that adolescent psychostimulant exposure in non-human primates *also* simplifies basal arbors in deep-layer prefrontal cortex (Selemon et al., 2007). A strong trend for a reduction in dendritic spine density was also reported by Selemon et al. (2007); similarly, oPFC dendritic spines are eliminated following adolescent cocaine exposure in the mouse (Gourley et al., 2012). The amygdala projects to deep-layer prefrontal cortex in both rodents and primates, with neurons terminating on dendritic spine heads of both apical and basal branches (Gabbott et al., 2006; Ghashghaei et al., 2007). Our findings thus indicate that the structural effects of adolescent psychostimulant exposure in critical cortico-amygdala circuits implicated in addiction (see Torregrossa et al., 2011) translate across rodent–primate species.

In tandem with arbor simplification, cocaine exposure impaired performance in an oPFC-dependent instrumental reversal task. In this task, mice are trained to nose poke for food reinforcers in a chamber with multiple response operandi. Once mice have acquired the reinforced response, the location of the reinforced aperture is “reversed,” in this case, from the lateral walls of the testing chamber to a center aperture, and mice must redirect responding to this previously non-reinforced aperture. Lesions of the lateral, but not medial, oPFC delay response acquisition, as does chronic cocaine exposure in adult mice (Krueger et al., 2009; Gourley et al., 2010). Conversely, instrumental reversal learning in drug-naïve mice is associated with subsequent cocaine self-administration patterns, with poor reversal performance predictive of higher rates of cocaine-reinforced responding (Cervantes et al., 2013). We report that even *subchronic* cocaine exposure in adolescent mice impaired response acquisition several *weeks* following drug exposure. Similarly, in a water maze reversal, early-adolescent cocaine exposure impairs response acquisition up to 10 days following exposure (Santucci et al., 2004). Together with multiple reports that cocaine exposure also occludes reversal learning based on stimulus–outcome associative contingencies (see Lucantonio et al., 2012), these findings highlight the long-term negative impact of cocaine on oPFC function, resulting in inflexible habit-like response strategies.

The oPFC also appears to regulate behavioral sensitivity and resilience to contextual stimuli associated with cocaine. For example, prolonged oPFC inactivation enhances context-induced reinstatement of cocaine seeking in rats, sparing drug-seeking behaviors induced by other conditioned stimuli (Fuchs et al., 2004; Lasseter et al., 2009). Thus, the *healthy* oPFC may gate the influence of contextual cues associated with drugs of abuse; repeated cocaine exposure could degrade this function through repeated stimulation of the dopamine D1 receptor, for example (Lasseter et al., 2014), simplification of neural structure (Figure 1), and/or imbalance between D1 and D2, given that D2 is highly expressed on basal arbors that were eliminated here (Brock et al., 1992).

### oPFC DENDRITE MORPHOLOGY IN DRUG-NAÏVE COCAINE-VULNERABLE MICE IS INTACT

Structural remodeling in the central nervous system is orchestrated by Rho family GTPases including RhoA (Rho), Rac1, and Cdc42, which coordinate the actin cytoskeletal rearrangements required for dendrite elaboration or simplification. Rho activation decreases branch extensions in multiple neural systems (e.g., Li et al., 2000; Wong et al., 2000), and interference with Rho activity promotes arbor growth (e.g., Sin et al., 2002; Couch et al., 2010) or activity-dependent remodeling of dendritic spines (Murakoshi et al., 2011). In the *adolescent* hippocampus, Rho activation causes dendritic arbor retraction, reducing overall length and complexity (Sfakianos et al., 2007).

Rho is inhibited endogenously by p190RhoGAP, which is activated by integrin receptor binding to extracellular matrix proteins (Arthur et al., 2000; Hernandez et al., 2004; Moresco et al., 2005; Bradley et al., 2006). *p190rhogap*-/- mice are not viable, and while *p190rhogap*+/– mice appear superficially normal, they exhibit significant vulnerabilities to genetic and chemical perturbations. For example, simultaneous heterozygosity for mutations in both p190RhoGAP and the cytoskeletal regulatory protein Arg kinase results in increased Rho activity and hippocampal dendritic arbor destabilization, accompanied by novel object recognition deficits (Sfakianos et al., 2007). Further, mice deficient in p190RhoGAP or the upstream effectors β1-integrin or Arg kinase are hyper-vulnerable to cocaine, generating a sensitization-like response following a single injection (Gourley et al., 2009, 2012; Warren et al., 2012).

The *p190rhogap*+/– mouse provides an opportunity to characterize neural morphology in an organism that is behaviorally vulnerable to cocaine *prior to cocaine exposure*. Throughout, however, we identified no differences in the size or complexity of excitatory deep-layer oPFC neurons between mutants and *p190rhogap*+/+ littermates. These findings suggest that although cocaine exposure remodels the same neuron population, *pre-existing* deficiencies in dendrite arbors do not obviously account for drug vulnerability. Previously, we characterized dendritic spine density on excitatory oPFC neurons in naïve *p190rhogap*+/– mice and *p190rhogap*+/– mice exposed to a subthreshold dose of the stress hormone corticosterone (Gourley et al., 2013b). Corticosterone reduced oPFC spine density in *p190rhogap*+/– mice, and these structural deficiencies emerged in concert with anhedonic-like behavior. Thus, p190RhoGAP may regulate the structural *response* of oPFC neurons to varied pathological insults. In line with this perspective, ethanol activates p190RhoGAP and thereby decreases actin stress fiber density in neonate astrocytes exposed to ethanol (Selva and Egea, 2011).

While we did not identify structural *predictors* of cocaine vulnerability in the oPFC, it is important to note that our findings do not preclude the possibility that pre-existing morphological or physiological characteristics in other models contribute to drug vulnerabilities. Additionally, pre-existing characteristics of cell populations in other brain regions—e.g., in the striatum, amygdala, or other regions of the frontal cortex (Ersche et al., 2012; Winhusen et al., 2013)—may significantly impact drug vulnerability even prior to active drug exposure.

## SUMMARY

The present results contribute to the general perspective that psychostimulant-induced neural remodeling has meaningful behavioral implications. These include potentially adaptive consequences. For example, cocaine-induced dendritic spine proliferation in the nucleus accumbens has been associated with behavioral resilience (e.g., Smith et al., 2014), and blockade of certain cocaine-induced dendritic spine modifications in the oPFC and nucleus accumbens can *increase*—*rather than occlude*—sensitivity to subsequent cocaine exposure (Toda et al., 2006; Pulipparacharuvil et al., 2008; Gourley et al., 2012). Meanwhile, the *correction* of long-term or metaplastic modifications following prolonged cocaine exposure may have behavioral benefits (Shen et al., 2009; Giza et al., 2013). Animal models provide an ideal venue to disentangle these issues, and to determine neurobiological vulnerability and resiliency factors using both prospective and retrospective approaches.

### Conflict of Interest Statement

The authors declare that the research was conducted in the absence of any commercial or financial relationships that could be construed as a potential conflict of interest.
